# Anti-Müllerian Hormone Concentrations in Women of Different Reproductive Age and the Chances of IVF Outcome: A Paradigm Shift is needed

**DOI:** 10.14336/AD.2024.1615

**Published:** 2026-01-20

**Authors:** Jakub Wyroba, Joanna Kochan, Liam Kelley, Sharif Iqbal, Paweł Kordowitzki

**Affiliations:** ^1^Malopolski Institute of Fertility Diagnostics and Treatment - KrakOvi, Krakow, Poland.; ^2^Fertility Disorders Clinic, Andrzej Frycz Modrzewski Krakow University, Krakow, Poland.; ^3^Department of Animal Reproduction, Anathomy and Genomics, University of Agriculture, Krakow, Poland.; ^4^Department of Cell Biology, Harvard Medical School, Boston, USA.; ^5^Division of Genetics, Department of Medicine, Brigham and Women’s Hospital and Harvard Medical School, Boston, MA, USA.; ^6^Department of Preclinical and Basic Sciences, Nicolaus Copernicus University, Torun, Poland.; ^7^Department of Gynecology including center of oncological surgery (CVK), Charite, Berlin, Germany.

**Keywords:** AMH, Anti-Mullerian Hormone, embryo, IVF, oocyte, FSH, reproductive aging

## Abstract

The study of Anti-Müllerian Hormone (AMH) has garnered considerable attention due to its critical implications in assessing and understanding both female and male fertility potential. Traditionally, AMH is recognized for its pivotal role in evaluating ovarian reserve and is a cornerstone in reproductive health assessments for women. The aim of this study was to challenge the traditional interpretation of AMH as a standalone predictor of IVF success. Through a retrospective analysis of 600 patients undergoing ICSI, we reveal that women with low AMH levels, traditionally classified as poor responders, can achieve unexpectedly high oocyte numbers, blastocyst formation, and pregnancy rates. This highlights the limitations of using AMH alone to predict IVF outcomes. Our findings advocate the importance of integrating additional factors, such as follicle-stimulating hormone (FSH), and the need for a more individualized approach to fertility treatment planning.

## INTRODUCTION

According to recent estimates, infertility affects around 8-12% of couples of reproductive age globally, with female factors accounting for approximately 30-40% of these cases. Identifying the underlying causes and addressing them through appropriate diagnostic and treatment methods are crucial for improving fertility outcomes. With important implications, women around the world increasingly postpone childbearing, leading to reduced fertility, more abortions, fetal abnormalities, and fewer children per family on average [1,2]. With advancing maternal age, the fertilized egg will exhibit lower quality and developmental competence, which contributes to increased chances of miscarriage due to several causes, such as aneuploidy, oxidative stress, epigenetics, or metabolic disorders. In consequence, women frequently go through depression or experience other psychological symptoms. One of the primary causes of female infertility is ovulatory dysfunction, which can be due to a variety of factors such as polycystic ovary syndrome (PCOS), premature ovarian failure, or hypothalamic-pituitary-ovarian axis disorders [3-5]. Therefore, *in vitro* fertilization (IVF) is a complex and rapidly evolving field of reproductive medicine that has revolutionized the way in which individuals and couples struggling with infertility can achieve their dreams of parenthood [6-8]. The development of IVF technology has provided hope to millions of people worldwide, offering a viable solution to a wide range of fertility challenges. Over the past three decades, IVF has steadily gained momentum in the United States [9]. This remarkable progress can be attributed to the continuous advancements in the various procedures and techniques involved in the IVF process, from the collection and handling of gametes to the culture and selection of the most viable embryos for transfer [10]. As the field of IVF continues to evolve, researchers and clinicians remain dedicated to exploring new avenues for improvement. However, in many cases, the adequate classification of women if they will have a good chance for a successful IVF remains elusive. Till today, the Anti-Müllerian Hormone (AMH) is used to provide patients with a prediction regarding the remaining pool of oocytes in the ovary [11]. The unparalleled ability of AMH to furnish consistent data throughout the menstrual cycle distinguishes it from other hormonal markers that are affected by cyclical variations, making it an invaluable asset in fertility evaluations and treatments. One of the main female factors deciding to undertake the IVF procedure is a low ovarian reserve, in other words, a low AMH value, and, consequently, poor ovarian response (POR) to stimulation and a small number of collected oocytes. It is well proven that ovarian reserve decreases with age, but there is also a group of younger patients with unexpected POR [12,13]. AMH is a member of the transforming growth factor-β family, which was first identified for its role in fetal sexual differentiation. It is produced by the granulosa cells of the ovarian follicle and has since been studied for its role in ovarian folliculogenesis and as a marker of the ovarian reserve [14-16]. AMH signals via the type II receptor (AMHRII), which leads to the recruitment of the type I receptor, resulting in the activation of SMAD proteins [17]. This leads to the regulation of specific target genes. AMH induces the recruitment of a small cohort of growing follicles from the primordial follicle pool. The global effect of AMH on the ovary is the inhibition of the cyclic recruitment of a group of primordial follicles and the growth of a group of small growing follicles [17]. Primordial follicles that are not exposed to AMH continue to recruit and grow to become dominant follicles, ovulate, and contribute to the *corpus luteum*. A negative feedback loop for AMH has also been identified in the ovary. This is an important concept in understanding the measurement of AMH from the ovary.

The interpretive power of AMH testing goes beyond simple measurement; it directly informs the strategic planning of fertility interventions. Among women undergoing fertility treatment, AMH levels serve as a critical guide for specialists aiming to design optimal stimulation regimens. Such personalized plans are essential as they can significantly affect the effectiveness of the treatment. AMH levels can indicate how well a patient might respond to ovarian stimulation, which is a vital component of procedures such as *in vitro* fertilization (IVF) [18-20]. AMH determination is now widely used to help achieve the best ovulation induction strategy for IVF with high success rates. The first evident rise in AMH levels in female serum occurs during the late stages of primary follicle development just prior to antral follicle formation. Levels then peak during the preovulatory phase with the onset of declining fertility. AMH then ceases to be produced immediately after menopause [21,22]. Consequently, the decrease in AMH levels also reflects a kind of biological clock for women. In clinical practice, ovarian reserve testing, besides the measurement of AMH values, includes antral follicle count (AFC). Both markers can provide valuable prognostic information to guide infertility management and treatment planning. These tests can help identify patients at risk of poor response or hyper-response to gonadotrophin stimulation, allowing clinicians to tailor treatment protocols accordingly. In our study, we aim to show that a low AMH level does not always go in parallel with a low oocyte retrieval number and IVF success. In our study, we aim to challenge the traditional interpretation of AMH as a standalone predictor of IVF success and to show that a low AMH level does not always go in parallel with a low oocyte retrieval number and IVF success.

## MATERIALS AND METHODS

**Figure 1.**
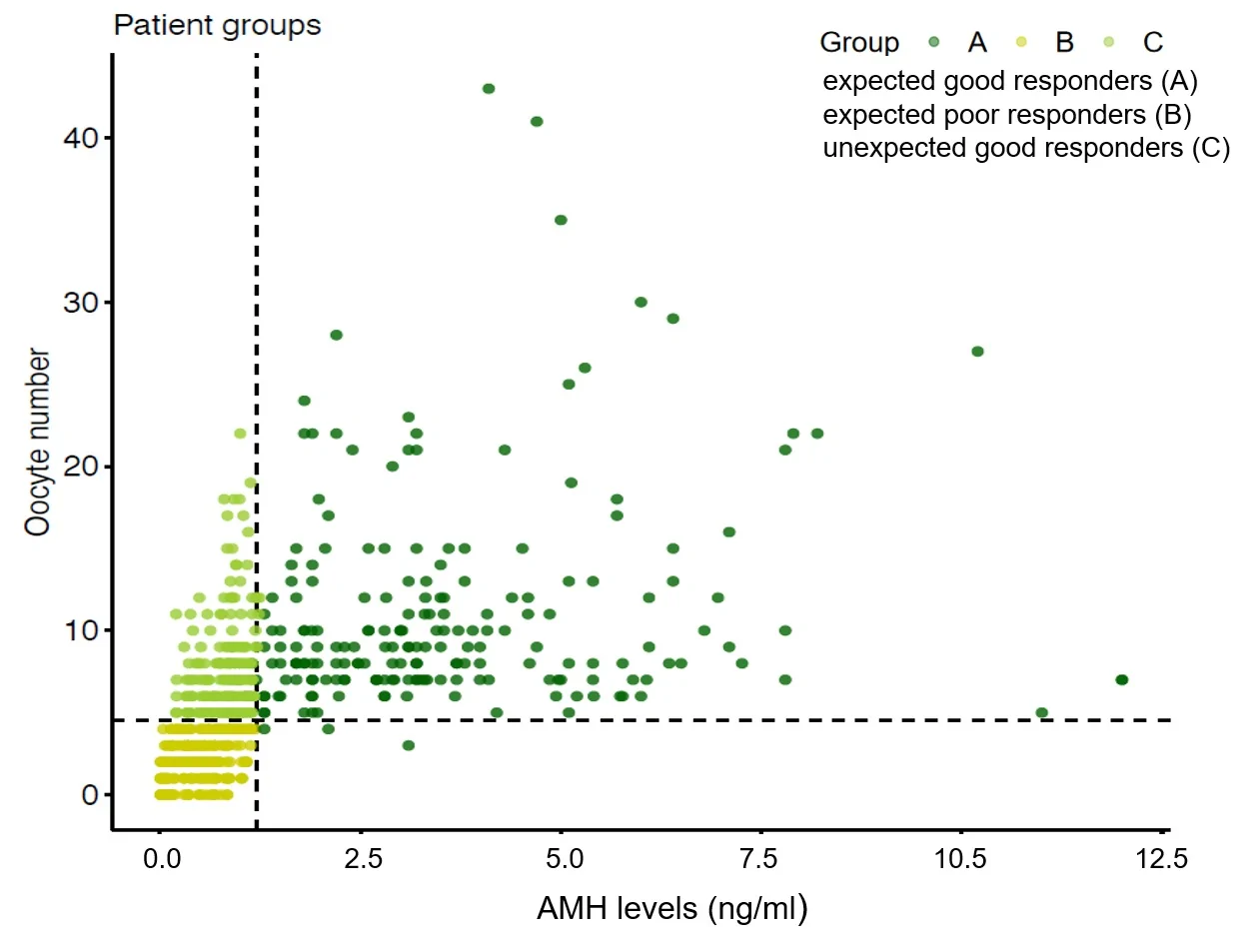
**Definition of patient groups**. Patient groups were delineated based on patient AMH level and oocyte number into expected good responders (A) n=207, expected poor responders (B) n=234, or unexpected good responders (C) n=202. No statistical methods have been used here since it is a descriptive figure.

This is a retrospective study of 600 patients who underwent intracytoplasmic sperm injection (ICSI) cycles at a Krakovi Clinic in Kraków (Poland) from June 2021 to June 2024. Institutional ethics committee approval (KBKA/06/O/2024) was obtained. For the classification of the poor responders, we used the Bologna criteria established by the European Society of Human Reproduction and Embryology (ESHRE) in 2011 [23]. According to these guidelines, at least two of the following three Bologna criteria had to be present for a patient classified as a poor responder: (1) Advanced maternal age (>40 years) or any other risk factor for POR; (2) A previous POR (generating ≤3 oocytes with a conventional stimulation protocol); (3) An abnormal ovarian reserve test, meaning either AFC less than 5-7 follicles or AMH value below 0.5-1.1 ng/ml. In our study, the patient groups were delineated based on patient AMH level and aspirated oocyte number into expected good responders (A), expected poor responders (B), or unexpected good responders (C) (Fig. 1). Further patient characteristics are presented in Table 1.

**Table 1:** Baseline characteristics of the patients.

	Poor responders		Unexpected good responders		Expected good responders	
	AMH<1,2ng/ml ≤4oocytes		AMH<1,2ng/ml >4 oocytes		AMH≥1,2ng/ml >4 oocytes	
	<**35Y** **n=86**	≥35Y n=114	<35Y n=92	≥35Y n=108	<35Y n=110	≥35Y n=90
**Age (years)** **mean ± SD**	31,85±3	40,1±4	32,7 ±3	39,8±3	32,3±3	39,2±4
**AMH ng/ml** **mean ± SD**	0,4±0,3^a^	0,4±0,2^a^	0,7±0,2^a^	0,6±0,1^a^	3,7±1,3^c^	3,2±1,1^c^
**Retrieved oocytes** **n (mean ± SD)**	180 (2±1,2)^a^	207 (1,8±0,9)^a^	754 (8,2±4)^c^	852 (7,8±4)^c^	1282 (11,6±5)^c^	909 (10,1±4)^c^
**Maturation rate** **n (%)**	113/180 (63%)^a^	126/207 (61%)^a^	512/754 (68%)^a^	562/852 (66%)^a^	1089/1282 (85%)^b^	754/909 (83%)^b^
**Fertilization rate** **n (%)**	81/113 (72%)^a^	86/126 (69%)^a^	363/512 (71%)^a^	393/562 (70%)^a^	936/1089 (86%)^b^	641/754 (85%)^b^
**Cleavage rate** **n (%)**	77/81 (95%)	78/86 (91%)	352/363 (97%)	381/393 (97%)	889//936 (95%)	602/641 (94%)
**ET day 3** **n (%)**	27/41 (64%)^a^	25/43 (58%)^a^	15/73 (20%)^c^	14/81 (17%)^c^	14/94 (15%)^c^	10/72 (14%)^c^
**ET day 5** **n (%)**	4/41 (10%)^a^	4/43 (9%)^a^	28/73 (36%)^c^	26/81 (32%)^c^	38/94 (41%)^c^	22/72 (31%)^c^
**FET** **n (%)**	10/41 (26%)^a^	14/43 (32%)^a^	38/73 (46%)^b^	41/81 (50%)^d^	42/94 (44%)^b^	40/72 (55%)^d^
**Implantation rate** **n (%)**	19/41 (46%)^a^	9/43 (21%)^d^	50/83 (60%)^c^	25/81 (31%)^b^	62/94 (66%)^c^	24/72 (33%)^b^
**Ongoing pregnancy** **n (%)**	17/41 (41%)^a^	8/43 (18%)^d^	44/83 (53%)^c^	21/81 (26%)^d^	55/94 (59%)^c^	18/72 (25%)^d^

Abbreviations: AMH - Anti-Mullerian hormone, ET- embryo transfer, FET- frozen embryo transfer, PGT-A - preimplantation genetic testing for aneuploidy, a:b, values with different superscripts within the same rows differ significantly (p < 0.05), Ongoing pregnancy was defined when the pregnancy had completed over 12 weeks of gestation with visible fetal cardiac activity. a:b, b:d values with different superscripts within the same rows differ significantly (p < 0.05), a:c, b:c, a:d, d:c - differ highly significantly (p < 0.001).

### Study design

*Inclusion criteria:* Based on the diagnostic criteria of diminished ovarian reserve, the inclusion criteria of patients need to satisfy at least two of the following three criteria 3: (1) AMH<1.2 ng/mL; (2) AFC<5 follicles; and (3) bFSH≥10 IU/L.

*Exclusion criteria:* Patients with any of the following conditions were excluded: (1) polycystic ovary or polycystic ovary syndrome; (2) chronic diseases, such as hypertension, cardiovascular disease, autoimmune disease, and abnormal hepatic and renal function; (3) other endocrine diseases, such as hypogonadotropin hypogonadism (HH) and hyperprolactinemia; (4) uterine malformation; (5) chromosome abnormality; (6) a history of recurrent spontaneous abortion; and (7) an active period of infectious diseases. Written informed consent for participation was required for this study in accordance with the national legislation and the institutional requirements.

### Clinical protocols

Patients were treated using either the long agonist protocol or the short antagonist protocol. The type of protocol used depended on the AMH level and the overall hyperstimulation risk.

### Long agonist protocol

Starting 1 week before the expected menses (cycle day 18-23), patients received the GnRH agonist, triptorelin (Decapeptyl, 1 mg/d, sc). After successful pituitary downregulation (when the serum estradiol (E2) levels were < 40 pg/mL), ovarian stimulation was commenced with a fixed daily dose of 150-300 IU recombinant follitropin alfa (rFSH, sc) with or without an additional 75-150 IU menotropin (hMG).

### Antagonist protocol

A GnRH antagonist Cetrorelix (Cetrotide, 0.25 mg/d, sc) or Ganirelix (0.25 mg/d), was administered, commencing when the largest follicle reached a diameter of 14 mm. rFSH/hMG was initiated on day 2-4 of the cycle. The agonist and antagonist protocols were continued up to and including the day of human chorionic gonadotropin (hCG) administration, which was when the leading follicle reached a diameter of 18 mm or more and at least three follicles reached a diameter of 17 mm or more. rFSH was then stopped, and a single sc bolus of 10,000 IU hCG (Eutrig) or 6,500 IU rhCG (Ovitrelle) was administered 36 h before the planned time of oocyte retrieval. When there was a risk of OHSS in an antagonist cycle, the trigger was a single sc bolus of triptorelin 2mg, and a freeze-all policy was applied. All follicles 12 mm or larger were aspirated. Subsequently, the oocytes were inseminated via ICSI, and a single embryo was transferred 3 or 5 days later. Luteal support in the form of intravaginal progesterone (Cyclogest, 400 mg twice a day) was administered starting from the day after oocyte retrieval until a serum pregnancy (b-hCG) test was performed 17 days later.

### Ovarian Stimulation Monitoring in ICSI

Baseline blood sampling and transvaginal sonography (TVS) was performed on day 2 or 3 of the pretreatment cycle for all patients. Monitoring of response during the treatment cycle consisted of TVS and blood sampling for hormonal analysis on cycle days: 2-3 (E2, FSH, LH); 5-6 (E2); 8-9 (E2); and day of hCG administration (E2, P4). Additional TVS monitoring was performed as clinically indicated.

### Preparation for Frozen Embryo Transfer

Treatment with oral E2 was started on the first, second or third day of the cycle to prime the endometrium and suppress spontaneous follicle growth. Oral estradiol was administered in an incremental fashion 2 mg/day during days 1-7, 4 mg/day during days 8-12, 6 mg/day during days 13 to embryo transfer. Usually, after 12 - 14 days of E2 administration, vaginal ultrasound examination was performed for endometrial thickness measurement and to confirm the absence of a dominant follicle. When the endometrial thickness was >7 mm, P4 supplementation was commenced, and the timing of FET was scheduled accordingly. For t-NC, TVS was performed on day 2 or 3 of menses to rule out any cyst or *corpus luteum* remaining from the previous cycle. Cycle cancellation was usually undertaken in cycles with serum P4 >1.5 ng/ml on day 2 or 3 of menses. Transvaginal ultrasonographic monitoring was usually started on day 8-10 and endocrine monitoring, measuring serum E2, LH and P4, was performed when the leading follicle attained a mean diameter of approximately 15 mm in diameter. Following frequent endocrine and ultrasonographic monitoring, on alternate days or daily, the day of ovulation was precisely documented to schedule the timing of FET.

### Laboratory protocols

ICSI was performed using a Nikon Eclipse CS100 microscope and an RI Integra 3 micromanipulator (Research Instruments, Germany), following the standard technique. Embryos were in vitro cultured in SAGE® medium (Origio, Denmark) in an atmosphere of 6.0% CO2, 5.0% O2, and balanced N2 at 37°C. Blastocysts were graded according to the Gardner scoring criteria (Gardner et al., 2000; Sakkas and Gardner, 2012) based on the degree of expansion, as well as inner cell mass (ICM) and TE morphology. Blastocysts were biopsied 120-124h after ICSI, using the same micromanipulator and microscope used for ICSI. The zona pellucida was perforated using an Octax® laser (Vitrolife, Sweden) for 250 μsec. The biopsied TE cells were washed with D-PBS and placed in 0.2 mL polymerase chain reaction (PCR) tubes for PGS referral to Igenomix Inc. (Spain) and analyzed by next-generation sequencing (NGS). Following the biopsy, blastocysts were incubated for 1.5 h in Sage®medium and then vitrified. Blastocysts were vitrified using Kitazato® media and the Cryotop device (Kitazato, Japan) according to the manufacturer’s protocol. Blastocysts were warmed in Kitazato media for a minimum of 1.5h before transfer and then placed in EmbryoGlue® medium (Vitrolife, Sweden) or in Sage® medium. More than 95% of transfers were performed in Embryo Glue® medium.

### Statistical analysis

Non-parametric data, such as differences in the percentage values between groups, were assessed by the Chi-Square test since we did not assume a specific distribution of the variables measured on nominal and ordinal scales. Parametric data were expressed as means ± SD and compared by two-way ANOVA. Differences were considered significant when the P-value was ≤ 0.05. The statistical analysis was performed using PQStat 1.6.2 (PQStat Soft, Poznan, Poland) and the R software, version 4.4.1 (R Project for Statistical Computing). Parametric data were expressed as means ± SD and compared by two-way ANOVA to draw conclusions about the populations from which the data were generated. The Mann-Whitney tests were performed using the function wilcox.test, which is built into R. The comparison of slopes was done using the built-in ANOVA function and the emtrends function from the package emmeans.

**Figure 2.**
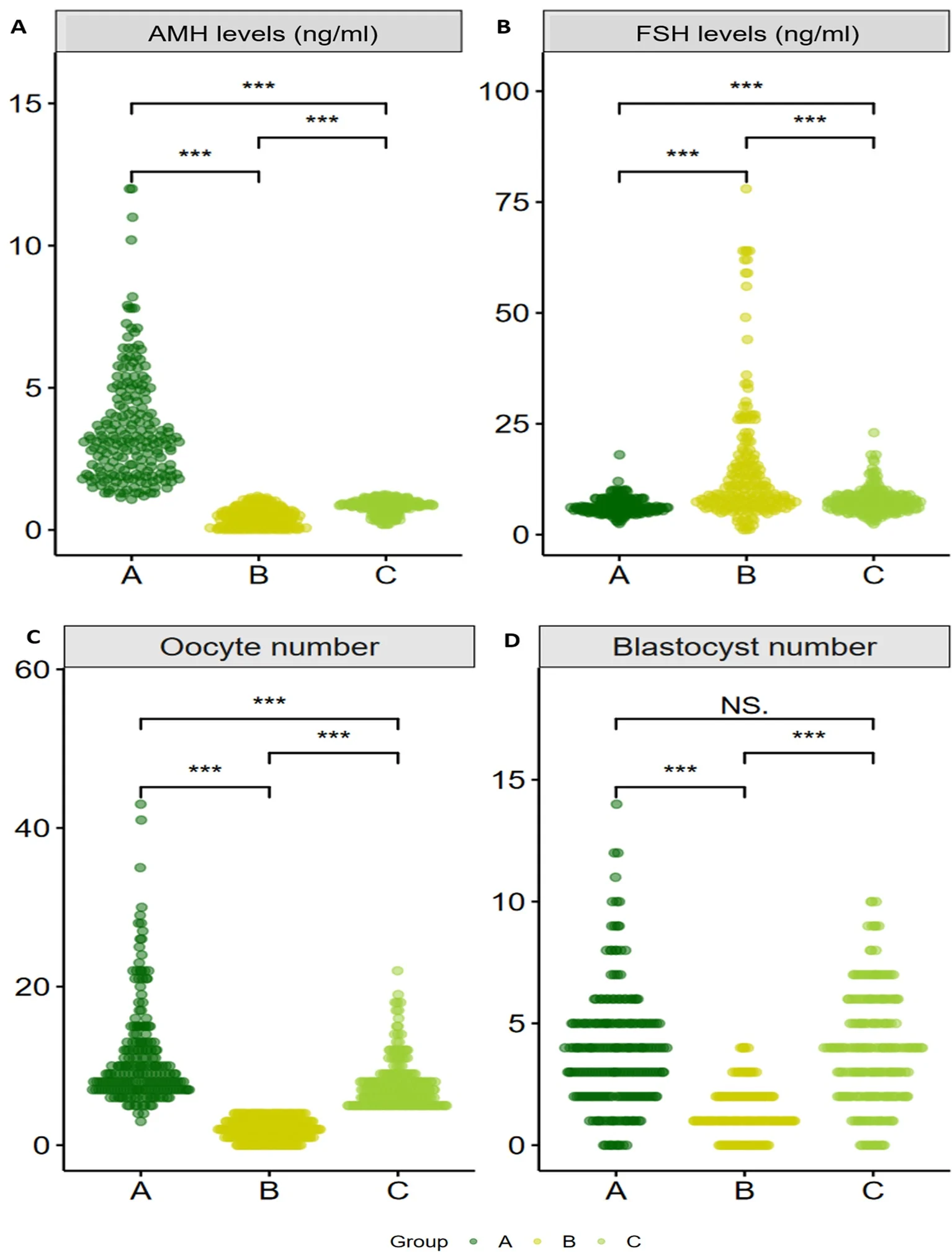
**Patient metrics, plotted by patient group**. Data, meaning AMH values (A), FSH values (B), oocyte number (C), and blastocyst number (D), are presented as violin-style density plots with individual points. Significance, when applicable, is shown between groups. Statistical significance was determined using the Wilcoxon rank-sum test. Significance: NS. p > 0.05, * p < 0.05, ** p < 0.01, *** p < 0.001. Numbers of included patients: expected good responders (A) n=207, expected poor responders (B) n=234, or unexpected good responders (C) n=202.

**Figure 3.**
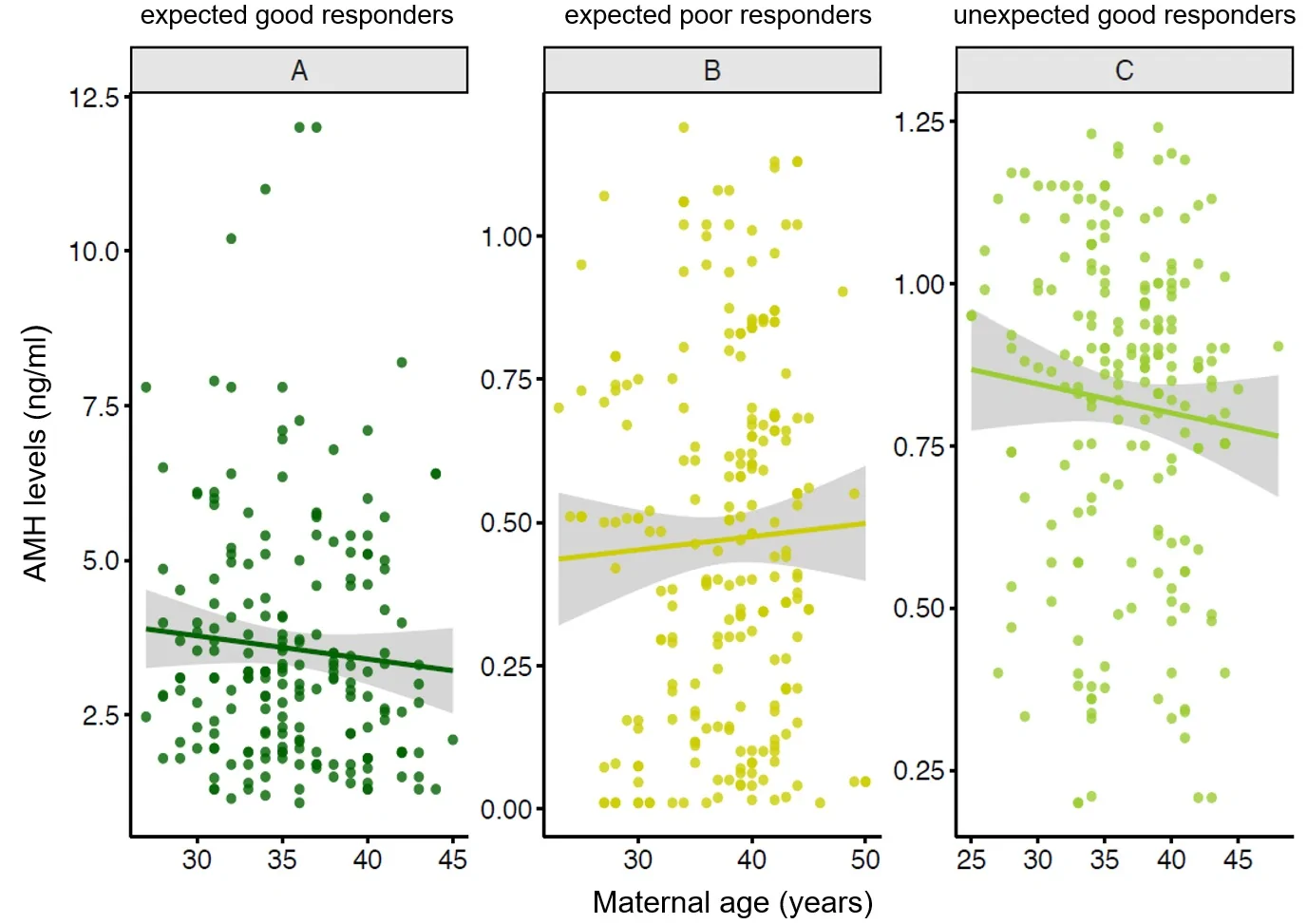
**Comparison of patient age to patient AMH level**. Data are divided into patient groups. Lines of best fit from linear regression are shown, with grey areas representing 95% confidence interval for each line of best fit. The comparison of slopes was performed using the built-in ANOVA function and the emtrends function from the package emmeans. Numbers of included patients: expected good responders (A) n=207, expected poor responders (B) n=234, or unexpected good responders (C) n=202.

## RESULTS

The application of AMH testing in clinical practice addresses the necessity of understanding individual ovarian biology, thereby enhancing fertility treatments' prognostic and therapeutic outcomes. Here, in our retrospective study, we analyzed 600 patients who underwent the expected ICSI cycles and respective hormonal treatments as described in the above protocols. According to their AMH concentration measured in blood samples and oocyte number on the day of ovum pick-up (OPU), we have selected 200 patients of different ages for our three groups, namely good responders (A), expected poor responders (B), or unexpected good responders (C). As shown in Figure 2, the AMH levels diver significantly (p < 0.001) between the groups analyzed by the Wilcoxon rank-sum test. In consequence, the oocyte number aspirated per OPU session was significantly different among the groups: In (group C: 8.1±4; group B: 2.1±1.2; group A: 10.1±5, p < 0.001, respectively). Interestingly, in group C there was a high number of aspirated oocytes per cycle, although the AMH was below <1,2ng/ml. Interestingly, there was also a significant difference in the level of FSH between all groups (Fig. 2).

In the next step, we compared the patients’ age with their AMH levels in the respective groups, as presented in Figure 3. In group A and group C, the AMH levels had a negative correlation with age, whereas in group B the AMH appeared to increase slightly with increasing maternal age, as presented by the linear regression analysis.

**Figure 4.**
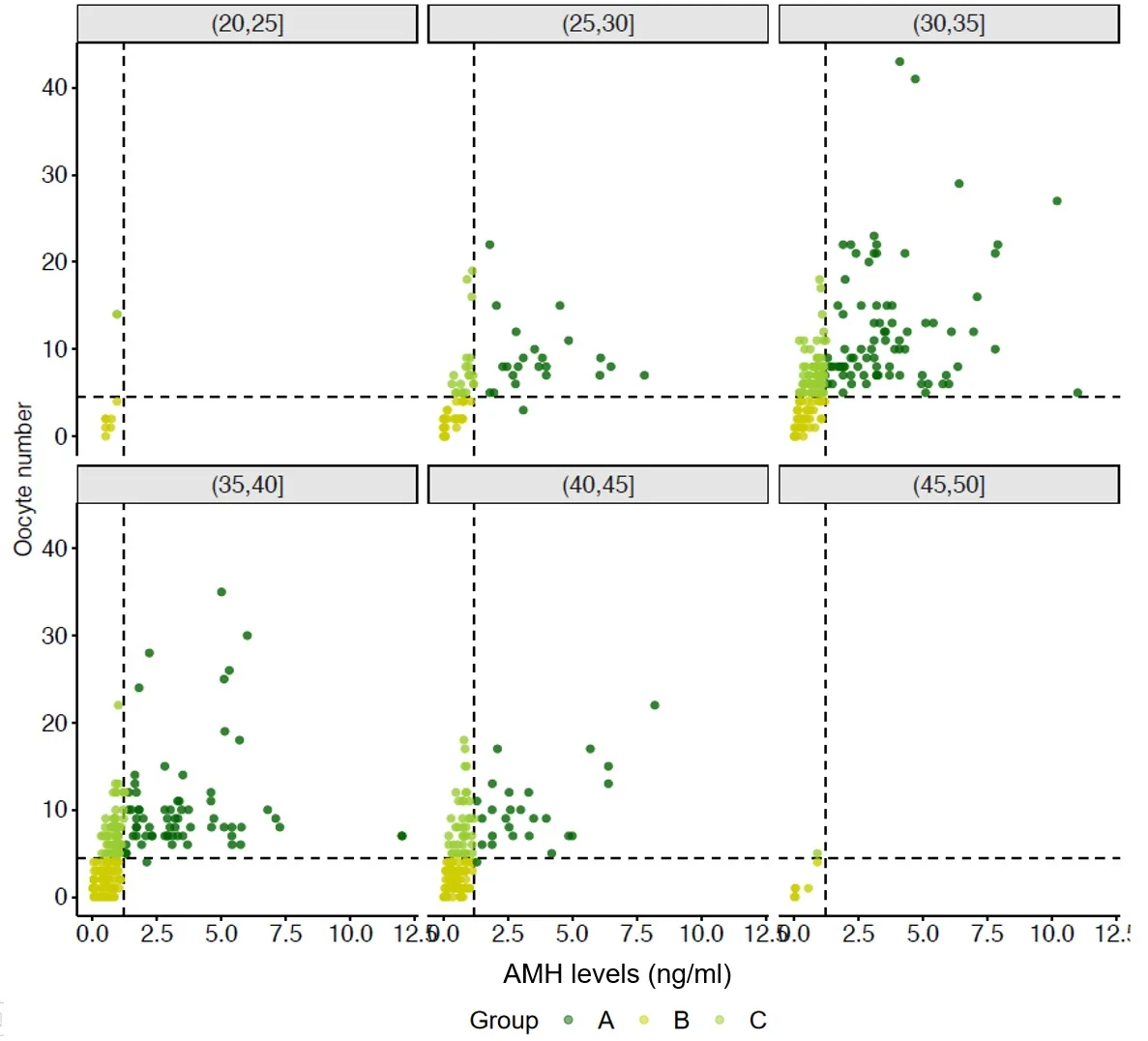
**Patient group abundance over time**. Patient groups were plotted by AMH and oocyte numbers separately for 5-year bins of patient age. The patient's age range is displayed above each plot, the numbers of included patients in each age range and group are as follows: range 20-25: n=0 in A, n=7 in B, n=2 in C; range 25-30: n=23 in A, n=29 in B, n=20 in C; range 30-35: n=85 in A, n=38 in B, n=66 in C; range 35-40: n=73 in A, n=82 in B, n=74 in C; range 40-45: n=26 in A, n=72 in B, n=39 in C; range45-50: n=0 in A, n=6 in B, n=1 in C. No statistical methods have been used here since it is a descriptive figure.

For a better analysis of the impact of AMH levels on oocyte number per aspiration/cycle with regard to maternal ageing, we decided to analyse the data in six subgroups, starting from the maternal age of 20 to 50 years, with 5-year age ranges in each subgroup. Noteworthy, there have been patients with a low AMH level and a low oocyte number although a young maternal age (20-25 years), as presented in Figure 4. This shows that a comprehensive understanding of AMH levels is, therefore, integral to customizing fertility treatment plans to align with the biological variations of each patient, regardless of maternal age.

As women age, various physiological changes occur that can significantly impact their reproductive health and fertility. One of the key hormones involved in this process besides AMH is the follicle-stimulating hormone (FSH), which plays a crucial role in the development of ovarian follicles and the maturation process of oocytes. Interestingly, in group C (unexpected good responders), the linear regression shows a positive trend, meaning with increasing maternal age, there is a slight increase in FHS levels, although the overall mean of FSH is low compared to group B.

Furthermore, a partial least squares (PLS) regression, a machine learning technique, has been performed to predict the oocyte number based on patients’ data (Fig. 6). After the PLS analysis on z-scored actual patient oocyte numbers, PLS regression coefficients were used to calculate predicted oocyte numbers across samples. The PLS regression clearly shows that group B stands out when compared to the other groups, showing a negative oocyte number prediction, which again emphasizes the specific characteristics of this group. It is worth noting that the PLS analysis also confirmed that the patients of group C have a comparable regression to group A, underlining that a low AMH does not always correlate with a low number of oocytes. This being said, AMH plays a pivotal role in the personalized approach to fertility treatment by allowing the tailoring of stimulation regimens according to the individual needs of each patient. In addition, variable loadings from partial PLS regression have been performed to predict oocyte number by multiplying each variable value (such as maternal age, AMH, and blastocyst number) by its loading weight and summing each variable to generate the predicted oocyte number z-score (Fig. 7). Here, we could show that AMH and blastocyst numbers show a loading above 0, meaning that a positive PLS weight can be interpreted to predict an increase in the oocyte number.

**Figure 5.**
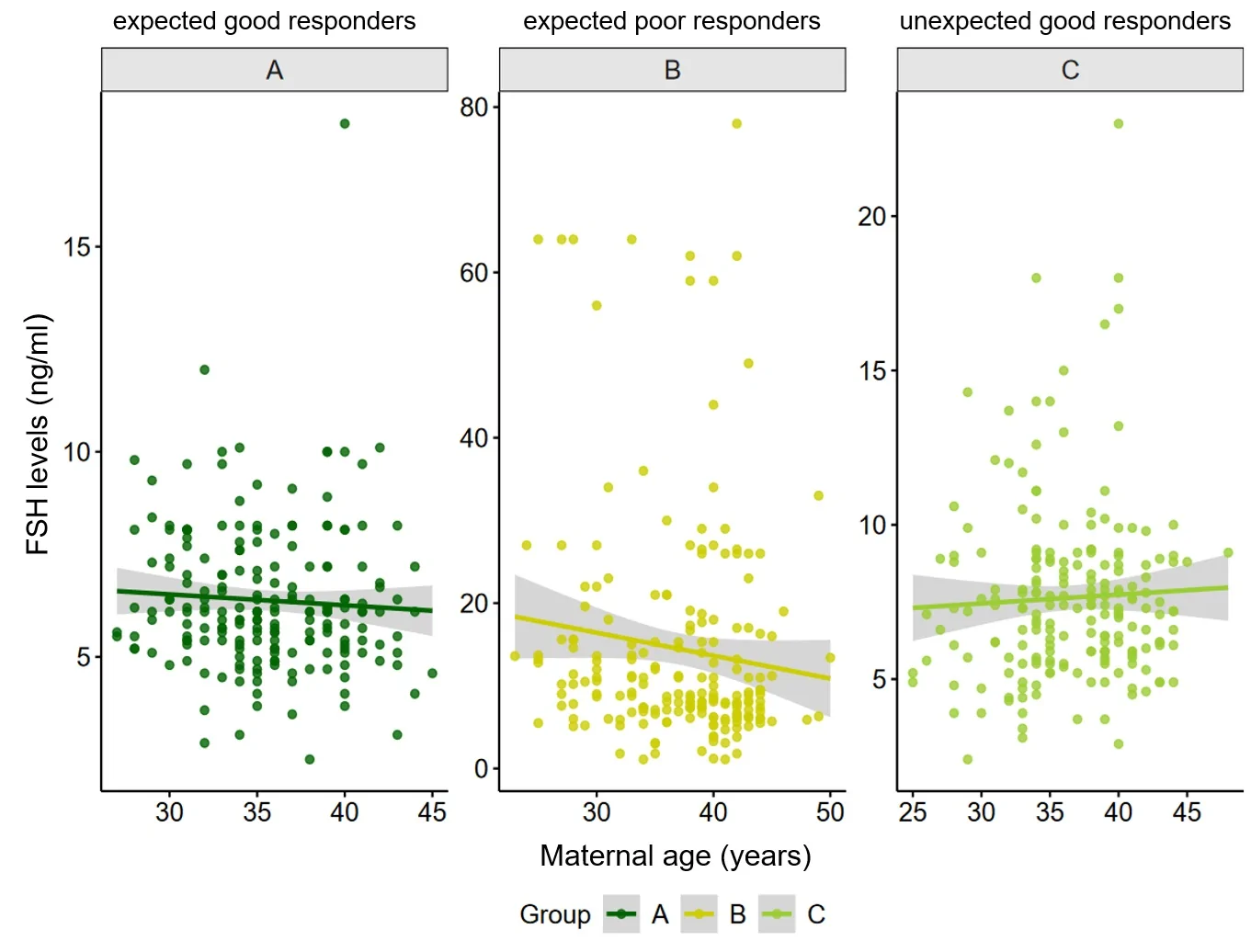
**Comparison of patient age to patient FSH level**. Data are divided into patient groups. Lines of best fit from linear regression are shown, with grey areas representing 95% confidence interval for each line of best fit. The comparison of slopes was performed using the built-in ANOVA function and the emtrends function from the package emmeans. Numbers of included patients: expected good responders (A) n=207, expected poor responders (B) n=234, or unexpected good responders (C) n=202.

Finally, the IVF success and pregnancy rate in all groups were analysed. As shown in Table 1, the fertilization and cleavage rate of generated oocytes did not differ significantly, whereas the percentage of good quality frozen blastocysts (Figure 8) which could be transferred into patients was significantly (p < 0.001) different between group B (26%) and group C (46%). Importantly, there was a significant (p < 0.001) divergence of the preimplantation genetic testing for euploidy of the generated blastocyst between group (5%) and group C (28%) (Figure 9). Figure 8 shows the effect of maternal age on blastocyst number. We could show that the blastocyst number in group C is comparable to that of group A, regardless of maternal age. However, in group B, there were significantly fewer blastocysts per patient than in the other two groups, which is also a consequence of the lower oocyte number in this group.

## DISCUSSION

Our study is unique with regard to the fact that women of group C who were classified as „unexpected poor responders” have shown a very good number of aspirated oocytes and IVF success, although having a low AMH value. Our analysis has shown that low AMH levels in women of advanced reproductive age do not necessarily mean poor chances of IVF outcome and pregnancy. This is particularly important as individualized treatment plans can vary significantly. Our results align with those presented in a similar recent study by Kovacs and coworkers [24], who have shown that the characteristics of our group B are comparable to the ones described for the low responders in their study.

**Figure 6.**
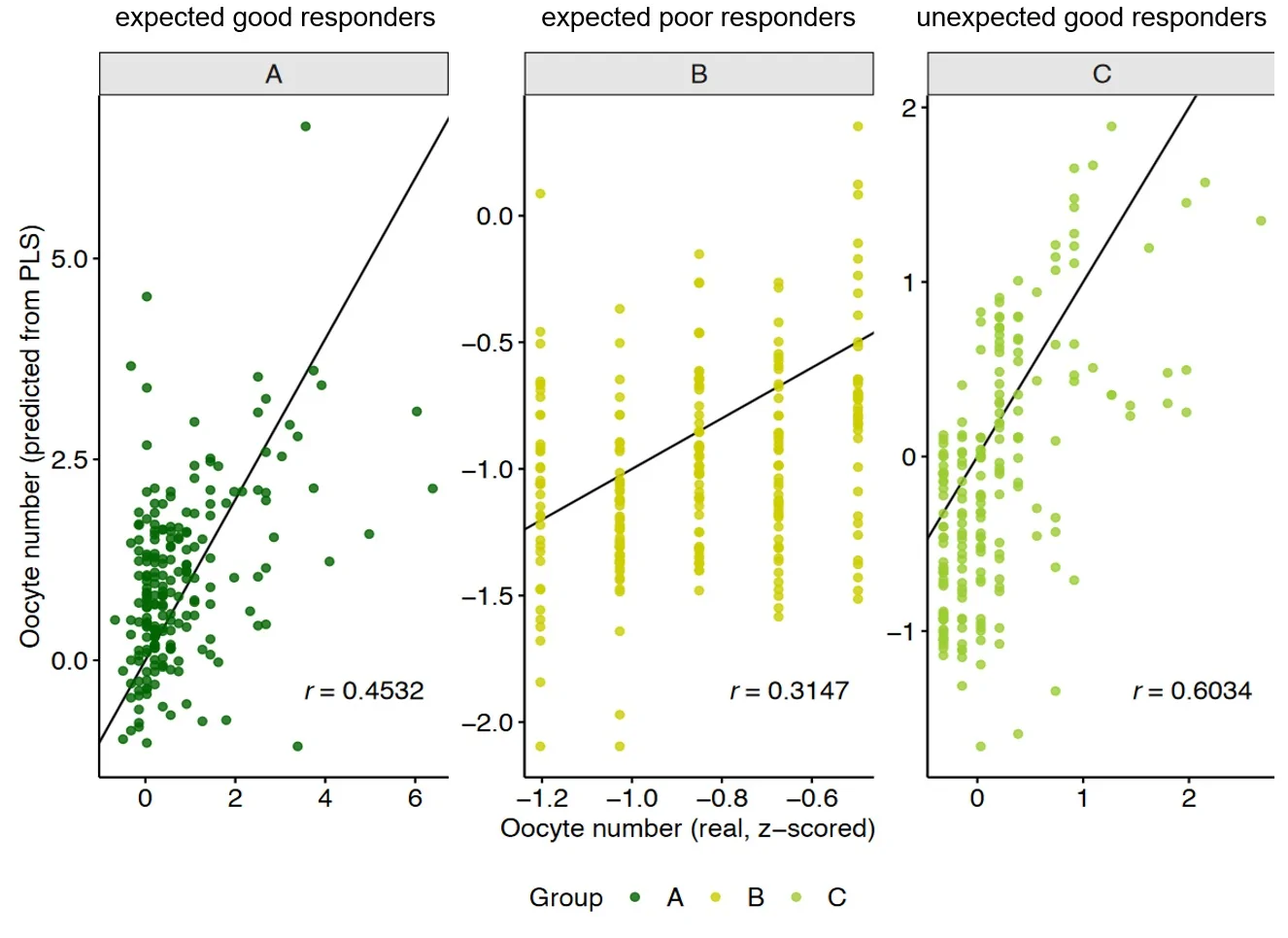
**Partial least squares regression for all groups**. Oocyte number prediction from partial least squares (PLS) regression has been used as a statistical test. After PLS analysis on z-scored actual patient oocyte numbers, PLS regression coefficients (r) were used to calculate predicted oocyte numbers across samples. Data are presented by group, with a line of slope one shown as a reference for perfect prediction. Numbers of included patients: expected good responders (A) n=207, expected poor responders (B) n=234, or unexpected good responders (C) n=202.

Another recent study has shown that a decreased ovarian reserve does not influence the conception rate [25]. Our study results clearly show that the old paradigm regarding AMH, meaning that AMH was believed to be a good predictor of the ovarian response and oocyte number, should not be used as an absolute discriminator in IVF clinics to classify a patient as a poor or good responder [26]. In clinical practice, as we have shown in Figure 2, the most valuable markers for predicting the ovarian response to hormonal stimulation during the IVF/ICSI procedure are AMH, FSH, AFC and the patient's age, although the prediction of expected ovarian response by any of the aforementioned values alone is not sufficiently reliable [27-29]. On this basis, physicians develop a strategy for the most appropriate therapeutic strategy for each patient and mentally prepare her for the likely outcome of the entire procedure. The starting point is to determine whether a patient will likely have a normal, poor or excessive response and select the ideal treatment protocol tailored to these predictions. A particularly difficult group of patients are those with poor ovarian response (POR), which occurs in approximately 9-25% of ART cycles [30,31]. However, despite the patient's classification as potentially being a POR based on AMH, FSH levels, and age, it happens that patients respond unexpectedly well to hormonal stimulation. Therefore, in our study, we shed light on these unexpected good responders compared to expected poor and good responders.

As a result of our research, we found that despite an unexpectedly good response and a satisfactory number of oocytes, statistically, there are more immature oocytes compared to the group of expected good responders (Fig. 2). Therefore, reduced ovarian reserve affects the quantity and quality of oocytes and their ability to mature. However, we found no differences between the groups' fertilisation and cleavage rates. There is no doubt that the evaluation of ovarian reserve is a crucial aspect in the management of infertility, and two key markers, AFC and AMH, have emerged as valuable tools in this assessment. However, it still remains elusive which of the two biomarkers, AMH or AFC, is more appropriate for assessing ovarian reserve.

**Figure 7.**
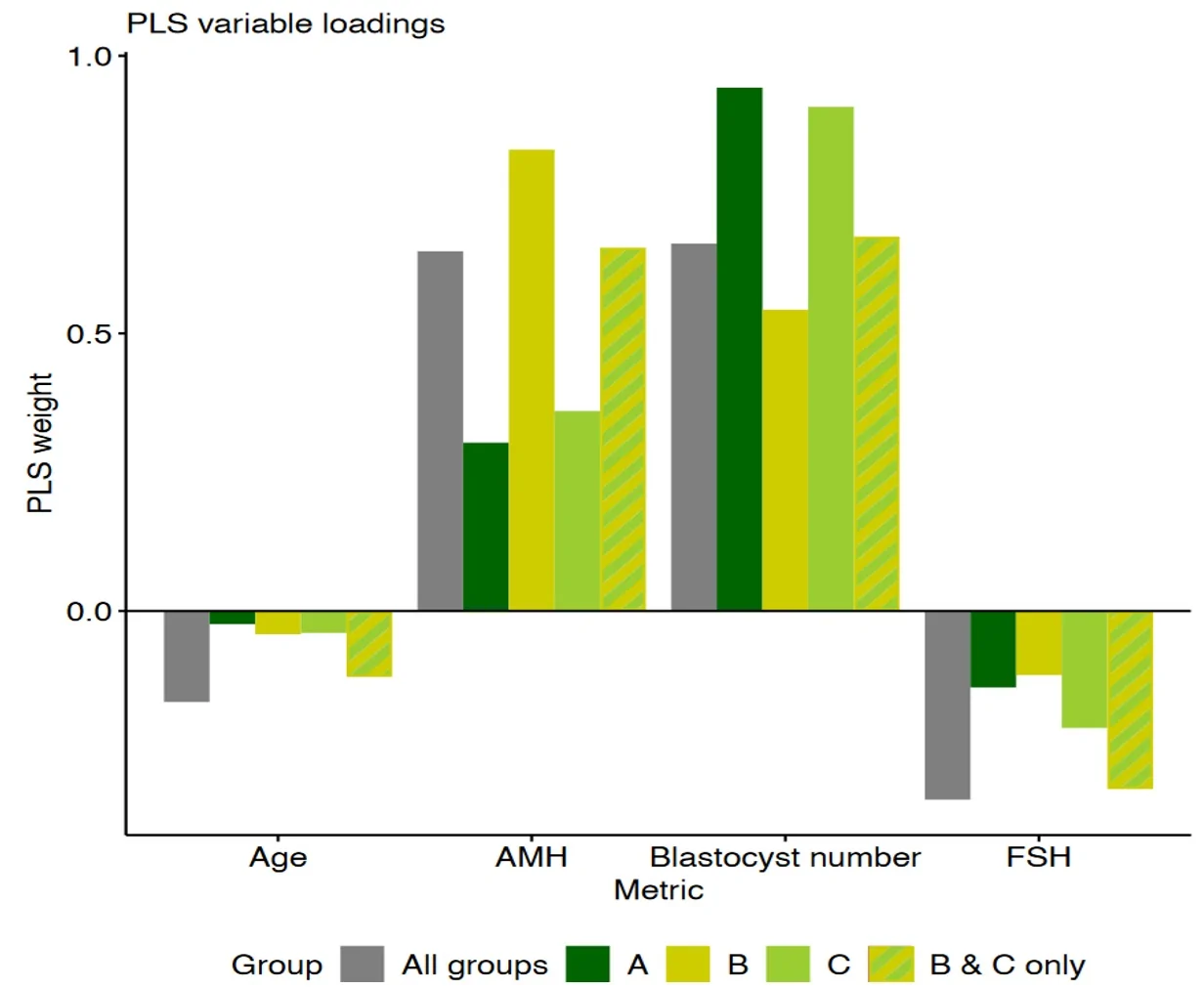
**Variable loadings from partial least squares (PLS) regression**. A loading above 0 denotes that an increase in the variable value, such as AMH level, is predicted to increase the oocyte number. Oocyte number predictions were generated by multiplying each variable value by its loading weight and summing each variable to generate the predicted oocyte number z-score. Data are presented for the models that used all groups, group A (expected good responders) only, or groups B (expected poor responders) and C (unexpected good responders) only. Numbers of included patients: expected good responders (A) n=207, expected poor responders (B) n=234, or unexpected good responders (C) n=202.

Moreover, both allow the prediction of natural menopause age, which is a correlative of ovarian reserve [32-34]. AFC and AMH have also been analyzed for their potential to tailor hormonal treatment strategies to elevate pregnancy outcomes [35-37]. Altogether, on the background of our study, we intend to emphasize that patients with an unexpected, good response, especially those at an advanced age, have a much greater chance of completing the IVF/ICSI procedure with the birth of a healthy child. This is of important relevance for clinics which are using the ESHRE guideline regarding ovarian stimulation for IVF/ICSI [38]. This guideline includes the recommendation to use either antral follicle count or AMH to predict the high and poor response of patients to ovarian stimulation. The patients who were included in this study also refer to the aforementioned guideline, which further recommends the use of the GnRH antagonist protocol over the GnRH agonist protocols in the general IVF/ICSI population but for predicted poor responders, the GnRH antagonists and GnRH agonists are recommended equally.

### Limitations

Our study was retrospective which may raise concerns such as selection and recall bias. Our Center specializes in the treatment of patients with POR and we have access to a unique, large group of patients with this disorder. This wide access to patients allowed us to select the most homogeneous research groups possible, eliminating confounding factors such as e.g. obesity or comorbidities. The only confounder factor is the male infertility factor, but in each study group, it was present at a comparable level ranging from 17 to 20%, which minimizes the bias. The advantage of our retrospective research is that it was based on data generated from only one IVF centre, and for all patients, the same protocols and procedures were applied, which ensured the repeatability of the procedures. However, our results should also be validated by other IVF centres to ensure further applicability in clinical settings. That is why we decided to publish these interesting findings and make them available to the research and clinical community.

**Figure 8.**
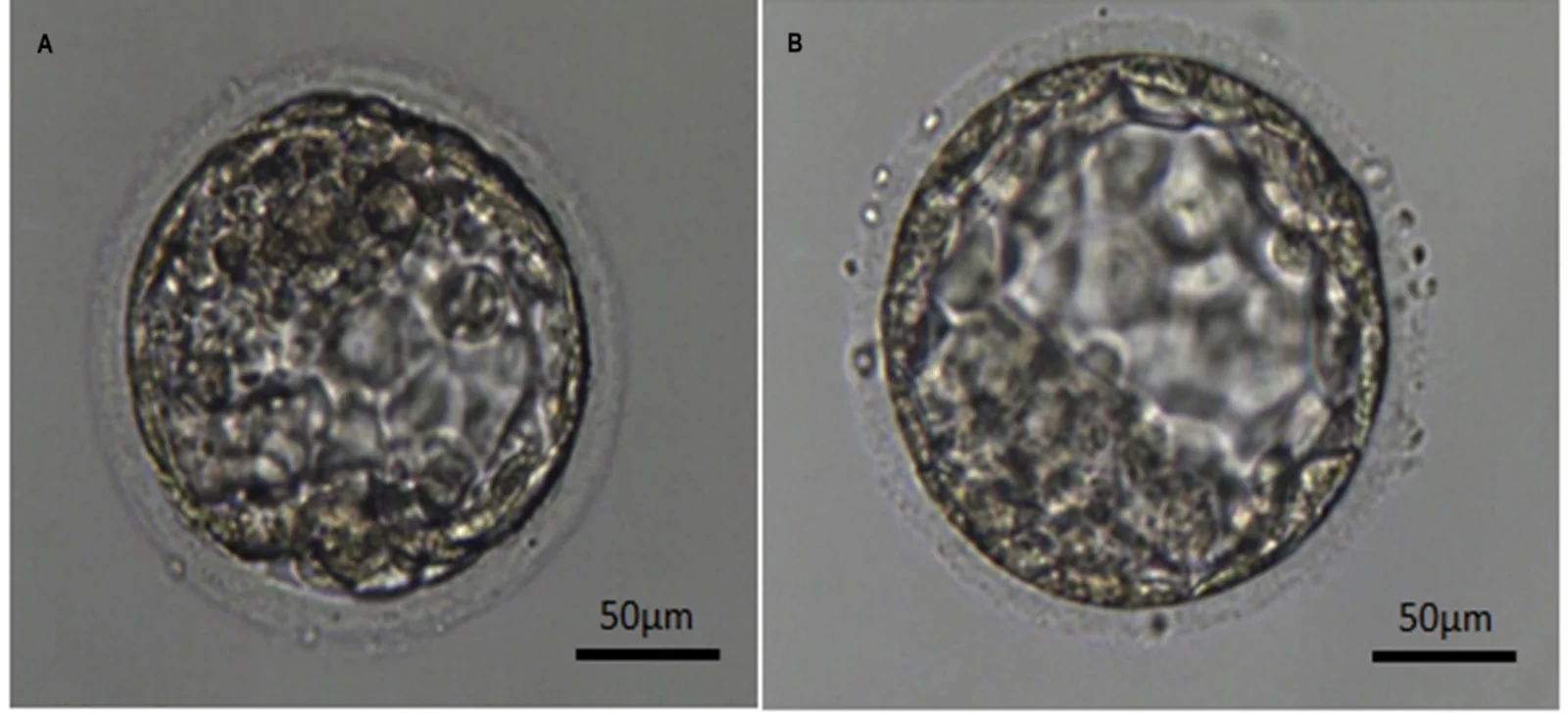
**Microscopic pictures of a day 5 blastocysts of group B**. The right panel B shows a representative microscopic photograph of a euploid blastocyst, and the left panel A shows an aneuploid blastocyst.

**Figure 9.**
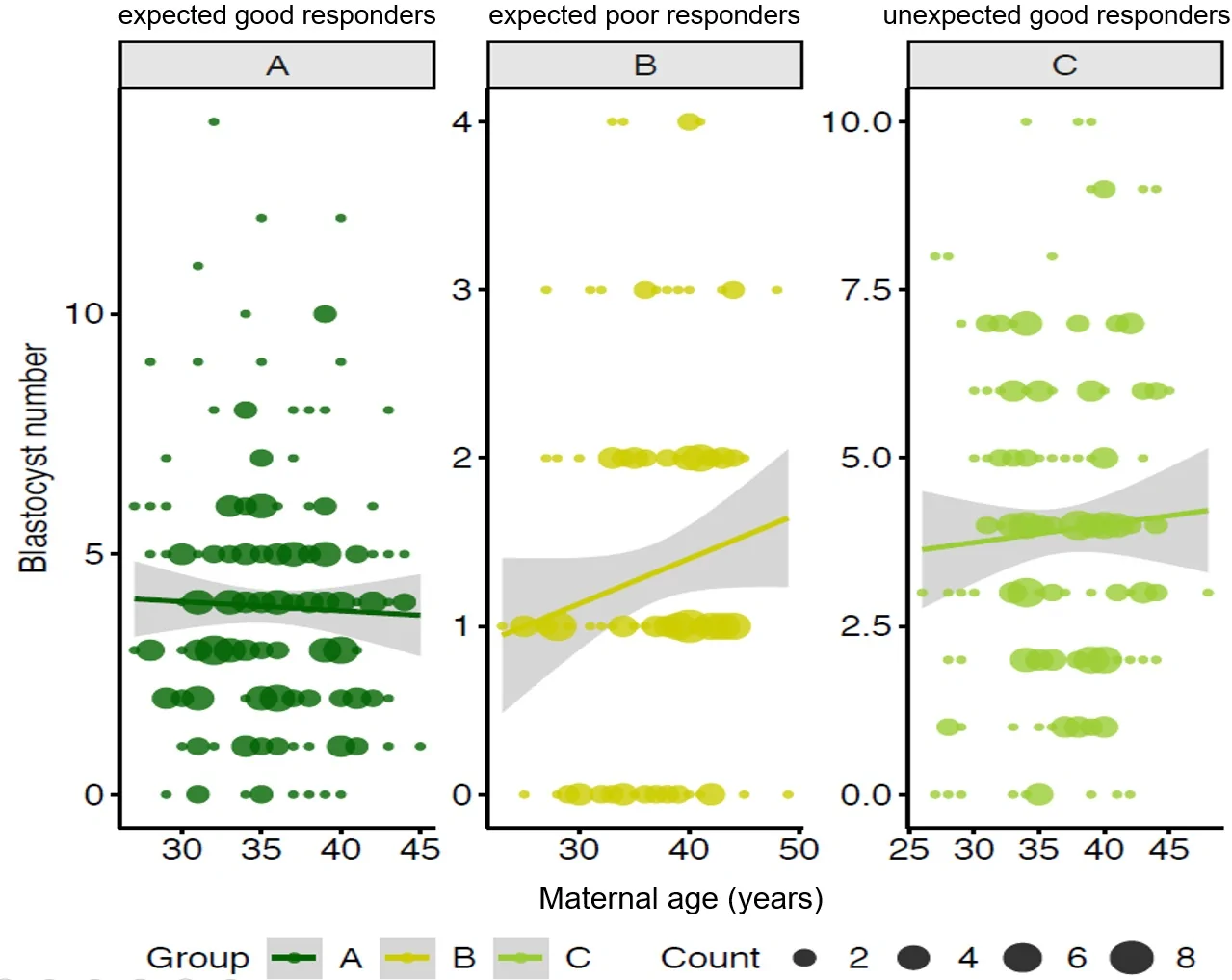
**Comparison of patient age to patient blastocyst number**. Data are divided into patient groups. Data are presented as circles of various sizes, where the circle size represents the number of data points at that particular location. Lines of best fit from linear regression are shown, with grey areas representing a 95% confidence interval for each line of best fit. The comparison of slopes was performed using the built-in ANOVA function and the emtrends function from the package emmeans. Numbers of included patients: expected good responders (A) n=207, expected poor responders (B) n=234, or unexpected good responders (C) n=202.

## Conclusion

In conclusion, the practical application of AMH extends beyond mere diagnostic use; it directly informs fertility treatments and interventions. By assessing AMH levels, healthcare providers can predict ovarian response to fertility medications, potentially influencing treatment protocols and patient counselling. This individualized approach is particularly beneficial in comprehensive fertility care, as it improves treatment effectiveness and aligns interventions with a woman's specific reproductive profile. Consequently, with our study, we aimed to underline the persistent and robust nature of AMH measurement, which enriches fertility evaluations, becoming a cornerstone in reproductive medicine. Overall, we can conclude that the application of AMH testing provides a strategic advantage in fertility treatment protocols. This approach not only maximizes the likelihood of successful pregnancy outcomes but also ensures the judicious use of medical resources. The precision of AMH-driven strategies in fertility care underscores its invaluable role in bridging the gap between scientific advancements and patient-centred care. Therefore, we conclude that a paradigm shift and further research on this interesting topic are needed to elucidate the intersection between AMH and IVF success.
